# Genomic Evidence Supporting a One Health Perspective on *Staphylococcus aureus* Bovine Mastitis

**DOI:** 10.3390/antibiotics15010098

**Published:** 2026-01-18

**Authors:** Gabriele Meroni, Valerio Massimo Sora, Giulia Laterza, Alessio Soggiu, Piera Anna Martino, Francesca Zaghen, Luigi Bonizzi, Luciana Colombo, Alfonso Zecconi

**Affiliations:** 1 Department of Biomedical, Surgical and Dental Sciences-One Health Unit, School of Medicine, University of Milan, Via Pascal 36, 20133 Milan, Italy; giulia.laterza@unimi.it (G.L.); alessio.soggiu@unimi.it (A.S.); piera.martino@unimi.it (P.A.M.); luigi.bonizzi@unimi.it (L.B.); alfonso.zecconi@unimi.it (A.Z.); 2 Department of Clinical and Community Sciences, School of Medicine, University of Milan, Via Celoria 22, 20133 Milan, Italy; 3 Associazione Regionale Allevatori della Lombardia, Via Kennedy 30, 26013 Crema, Italy; l.colombo@aral.lom.it

**Keywords:** *Staphylococcus aureus*, mastitis, One Health, whole genome sequencing, antimicrobial resistance

## Abstract

**Background/Objectives:** *Staphylococcus aureus* is a multifaceted pathogen responsible for diseases in humans and in several animal species, including dairy cows. This study aimed to characterize and compare the genetic diversity, lineage distribution, and antimicrobial resistance profiles of *S. aureus* isolates from bovine milk with human-derived reference genomes to investigate host adaptation and inter-species transmission. **Methods**: Genomic analyses were performed on *S. aureus* isolates from quarter milk samples of dairy cows together with human-derived sequences from public databases. Whole-genome sequencing and multi-locus sequence typing (MLST) were used to determine sequence type (ST) distribution, and the presence of key antibiotic resistance genes and mobile genetic elements (MGEs) was assessed. Comparative genomics was applied to evaluate gene content, phylogenetic relationships, and lineage–host associations. **Results**: The dataset encompassed bovine-adapted lineages (CC97, CC133, CC151) and human-associated lineages (CC1, CC5, CC8, CC30, CC45), as well as livestock-associated ST398 in bovine samples and human-adapted ST5 and ST6 in animals. ST8 was the only ST shared between animal and human isolates and showed differing resistance profiles, with animal ST8 carrying resistance determinants absent from human ST8. Bovine-adapted strains were characterized by recurrent loss of human-associated virulence genes and acquisition of bovine-associated mobile genetic elements, and *blaZ* and *mecA* were rarely detected in bovine-adapted CC97 but were frequently present in human CC5 and CC8. Overall, animal isolates carried fewer resistance genes than human isolates. **Conclusions**: *S. aureus* from dairy cows and humans displayed substantial genetic diversity, with evidence of host-associated lineages and dynamic changes in gene and mobile element content. These findings support the need for integrated One Health surveillance to track shared and host-adapted lineages and their antibiotic resistance determinants.

## 1. Introduction

*Staphylococcus aureus* is known for inducing various diseases in both animals and humans. In humans, it can induce skin infections, pneumonia, and more severe diseases such as endocarditis and toxic shock syndrome [1]. The rise in antibiotic-resistant clones, especially methicillin-resistant *Staphylococcus aureus* (MRSA), has intensified worries about public health and zoonotic transmission for this pathogen. In dairy cows, it is a principal causative agent of mastitis, resulting in a significant negative economic impact on the dairy industry [2]. Despite the extensive literature on the epidemiology and control of intramammary infections due to *S. aureus* [3,4,5,6], these infections are still recorded in many countries, and particularly in Italy [7,8,9].

The potential role of milk as a source of food-poisoning due to *S. aureus* enterotoxins is well known [10], as is its role in the case of livestock-associated methicillin resistance [11]. However, the potential zoonotic role of *S. aureus* commonly associated with intramammary infections in dairy cows is still unclear. This pathogen has a vast array of virulence factors and antimicrobial resistance genes [12,13,14,15,16,17] and its genetic apparatus suggests that the potential zoonotic capabilities should not be ignored.

The genetic diversity of *S. aureus* is evident in its multi-locus sequence typing (MLST), which classifies the bacterium into various sequence types (STs) and clonal complexes (CCs) [18]. Several STs are notably shared across humans and animals, revealing a complex global health interaction and making a One Health approach relevant. ST398 is primarily linked to livestock, mainly pigs, although it has also been detected in humans, particularly those with close contact to these animals [19]. Likewise, ST5 and ST6 have been identified in both meat products and human clinical isolates, indicating that foodborne transmission pathways may contribute to the epidemiology of *S. aureus* infections [20]. Besides cattle, wildlife has also been involved in the transmission dynamics of *S. aureus* with ST49 and ST581 being the main STs sharing genetic similarities with human isolates [21,22]. The existence of these shared STs prompts enquiries into the biological mechanisms that enable their persistence among various species. Bovine-adapted *S. aureus* lineages include CC97, CC133, and CC151 while human-adapted lineages include CC1, CC5, CC8, CC30, and CC45 [5]. *S. aureus* host spillover followed by adaptation to a new host is typically associated with the acquisition of a new set of genes specific for survival in the new host losing virulence to the former host species [23]. Recent comparative genomic reports indicate that bovine-adapted strains swiftly discard genes associated with human infections, thereby likely enhancing their fitness in bovine hosts [5,23,24]. Moreover, cows serve as a reservoir for antimicrobial-resistant (AMR) *S. aureus*, which could be transmitted to people and persist within them [12,13]. Few studies have tried to understand this association by correlating AMR and lineage. Few STs are known to be positive for *blaZ* or *mecA* among the *S. aureus* CC97 isolated from cows, which comprises many STs (ST97, ST115, and ST352). Moreover, ST5 (CC5), ST8 (CC8), and their variants are human-adapted lineages that have been isolated from cows and were positive for *blaZ* and *mecA* [25,26,27].

Despite its significance in human and veterinary medicine, research is scarce on the gene profile, strain-to-strain relationships, disease–host relationships, and host–pathogen interactions of *S. aureus*. We implemented genome-wide analyses in the present study to gain a more thorough understanding of these issues by comparing *S. aureus* strains isolated from the milk samples of dairy cows and human-derived sequences from public databases.

## 2. Results

### 2.1. Genomic Features of the Analyzed Strains

We performed Nanopore Whole Genome Sequencing on 50 *S. aureus* strains isolated from bovine milk. All the sequences were deposited in NCBI under the Bioproject PRJNA1243330. Table 1 summarizes the main descriptors of the sequenced strains. The distribution of *agr* types, SCCmec types, and sequence types (STs) among human (H_) and animal (A_) isolates reveals distinct population structures characterized by specific host-associated predominance. Regarding the *agr* locus, human isolates are predominantly *agr* group 1 (66%), followed by group 2 (30%), and group 3 (4%). Animal isolates show a similar pattern with slightly less dominance of group 1 (64%), group 2 (26%), and a higher proportion of group 3 (10%). The SCCmec typing, highlights the absence of methicillin resistant strains in the A_ cohort (98%), with the detection of a single strain found positive for *mecA* gene and clustered as SCCmec type V:Vc. In contrast 36% of H_ strains carried SCCmec IV:IVj type, 12% II:IIa and IV (other subtypes found comprise SCCmec types IV:IVa (6%), I:Ia (6%), as well as IV, V, and III (each 2%)).

Sequence type analysis further emphasizes the circulation of distinct clones in humans and animals. ST22, a lineage globally recognized as a hospital-associated MRSA strain linked to epidemic spread and high virulence, is the most represented ST in H_ strains (38%). Other human-associated STs include ST5 (14%), ST8 (10%), and additional less frequent STs such as 228 (6%), 105 (4%), 1 (4%). In A_ isolates, ST8 is the most frequent (16%), closely followed by ST352 and ST151 (14%), ST700 (8%), and other STs such as 71, 1094, 6, 6103, 701, 504.

### 2.2. Pangenome Analysis

**Figure 1:**
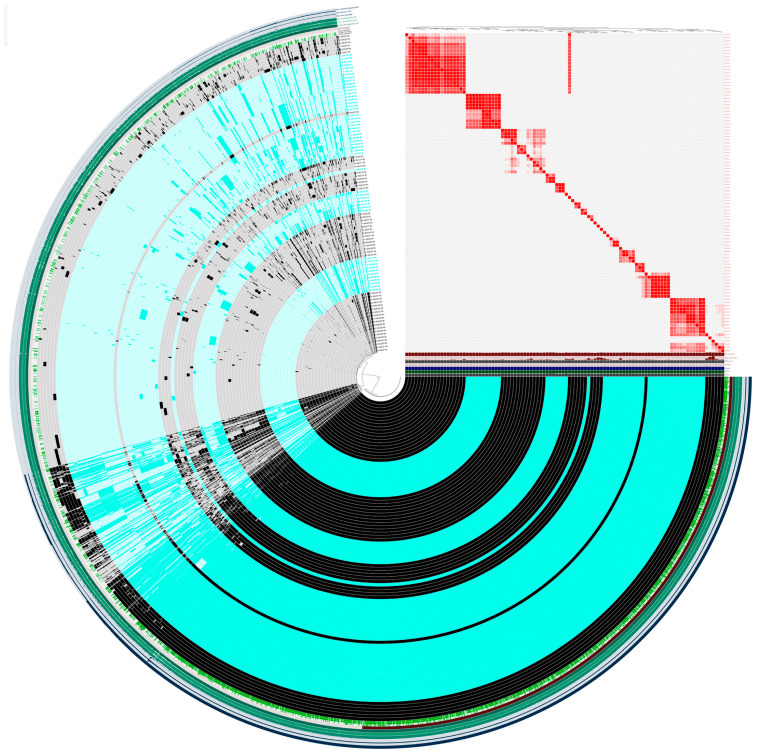
Pangenome structure and host-associated genomic clusters. Circular representation of the pangenome generated with the Anvi’o pipeline, in which each ring corresponds to a single genome and each radial sector to a gene cluster, with dark segments indicating gene presence and light segments gene absence. Strains of animal origin are highlighted in blue and strains of human origin in black, allowing visual identification of host-associated clusters of genomes with similar gene content. The outermost colored rings depict categorical genome metadata, where distinct shades of green correspond to different annotation or bin categories assigned in the Anvi’o interface (for example, separate genome groups or annotation sources) rather than to a quantitative gradient. The inset matrix displays pairwise average nucleotide identity (ANI) based on gene presence–absence at a 99% similarity threshold, where red blocks denote highly similar genomes and lighter regions indicate greater genomic distance, revealing clades of closely related strains that mirror the host-associated structure of the pangenome.

Figure 1 (see also Supplemental Figures S1–S4) illustrates the pangenome generated with the ANVI’O pipeline.

The pangenome comprised 7200 gene clusters, including 1729 core genes (found in at least 99% of strains), 157 soft-core genes (present in 95–99% of strains), 1352 shell genes (in 15–95% of strain), and 3962 cloud genes found in less than 15% of strains. The dominance of cloud genes, which represent more than half of the pangenome, together with a sizeable shell fraction, is consistent with an open pangenome and extensive genomic flexibility. The separation between core and soft-core components highlights a conserved backbone of housekeeping and essential functions, accompanied by a subset of quasi-essential genes whose incomplete distribution suggests ongoing gene gain and loss dynamics. This soft-core fraction likely reflects adaptation to specific ecological contexts or hosts, where certain functions provide a selective advantage without being strictly required across all lineages.

The accessory genome (shell and cloud) accounts for the majority of gene clusters and underscores marked variability among strains, with many genes present in only a limited number of strains, thus indicates a high genomic plasticity and suggests that horizontal gene transfer and niche-specific selection are important drivers of species evolution.

Clustering patterns based on gene presence–absence and average nucleotide identity (ANI) revealed groups of highly similar genomes, often corresponding to strains of shared animal or human origin. These clusters indicate that host-associated lineages tend to retain more similar gene clusters, supporting the contribution of host adaptation to the shaping of the accessory genome.

The combination of a stable core and a highly dynamic accessory genome likely facilitates persistence in different hosts and environments, and may underpin differences in virulence, colonization ability, or antimicrobial resistance potential between animal- and human-derived strains.

### 2.3. Phylogeny

**Figure 2:**
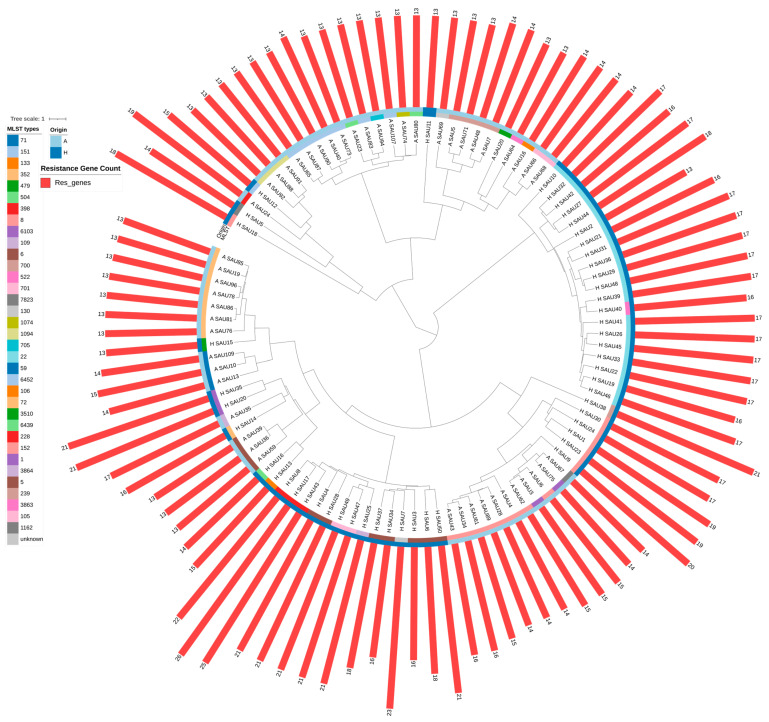
Phylogenetic relationships and antimicrobial resistance gene profiles across animal and human strains. A circular phylogenetic tree in which each leaf represents a single isolate, with animal-derived strains color-coded in blue and human-derived strains in black. Branch lengths reflect evolutionary distance, and the clustering of strains along the tree reveals both host-specific lineages (forming monophyletic clades of the same origin) and mixed clades containing both animal and human isolates, indicating a complex evolutionary history with varying degrees of genetic isolation and admixture between populations. Radial bars extending from each tip represent the number of antimicrobial resistance genes per genome, detected using the CARD database and scaled to allow visual comparison of resistance gene burden across isolates. Red bars are scaled to highlight variation in resistance gene content, with human-derived strains typically showing greater bar length than animal-derived strains, reflecting the significantly higher mean resistance gene count in clinical versus zoonotic isolates.

Whole genome phylogenetic reconstruction (Figure 2) revealed substantial genetic diversity, with animal- and human-derived strains distributed across distinct branches and, in several cases, clustering within shared clades. A complex evolutionary pattern is indicated by the existence of both host-specific lineages and mixed clades. This pattern is marked by separate host-associated divergence in some lineages, while other lineages exhibit indications of genetic mixing or recent zoonotic trade.

A difference in accessory gene acquisition emerged between the two populations; human-derived isolates harbored significantly more resistance genes (mean 17.9 per genome) compared to animal-derived strains (mean 13.8 per genome; Welch’s *t*-test, *p* < 0.0001). This pattern suggests that human-associated lineages have accumulated antimicrobial resistance determinants, consistent with exposure to antimicrobial selective pressure in clinical and community settings. Conversely, animal-derived strains displayed a higher mean count of virulence-associated genes (77.02 vs. 74.64; Welch’s *t*-test, *p* = 0.0043), suggesting that preservation of a broader virulence factor repertoire may be maintained through ecological or infection-related selection in animal hosts.

These results indicate that whilst a conserved phylogenetic backbone links the species, differential acquisition and dissemination of resistance and virulence genes reflects distinct selective pressures ongoing in animal and human hosts, with implications for pathogenic potential and treatment challenges in each population.

### 2.4. Antibiotic Resistance of S. aureus in Human (H_SAU) and Animal (A_SAU) Strains

**Figure 3:**
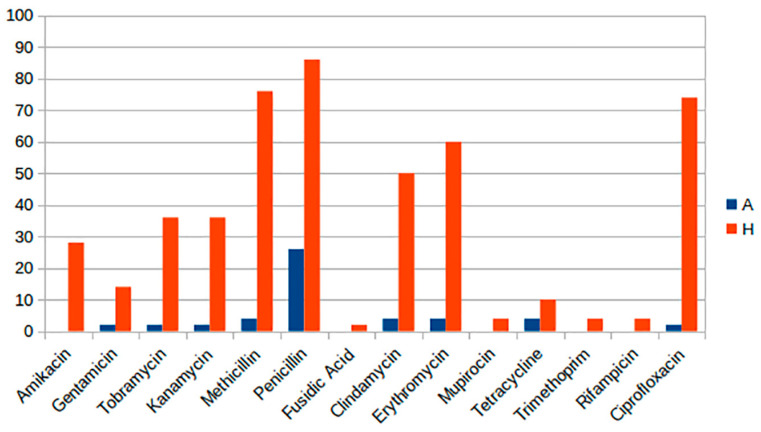
Predicted antibiotic resistance expressed as proportion (%) in H_SAU and A_SAU strains.

Figure 3 summarizes the predicted phenotypic antibiotic resistance detected in the two groups (H_SAU and A_SAU).

Predicted antibiotic resistance distribution reveals substantial disparities, with increased resistance in H_SAU across all examined antibiotic classes. Specifically, amikacin resistance is absent in animal strains, whereas it is present in 28% of human strains. Resistance to aminoglycosides (gentamicin, tobramycin, and kanamycin) in human strains is 14%, 36%, and 36%, respectively, although in animal strains, it ranges from 2% to 4%. Significant disparities are noted with methicillin (4% A vs. 76% H), penicillin (26% A vs. 86% H), erythromycin (4% A vs. 60% H), and ciprofloxacin (2% A vs. 74% H). Certain antibiotics, including fusidic acid, mupirocin, trimethoprim, and rifampicin, exhibit no resistance in animal strains, but human strains demonstrate resistance rates ranging from 2% to 4%.

**Figure 4:**
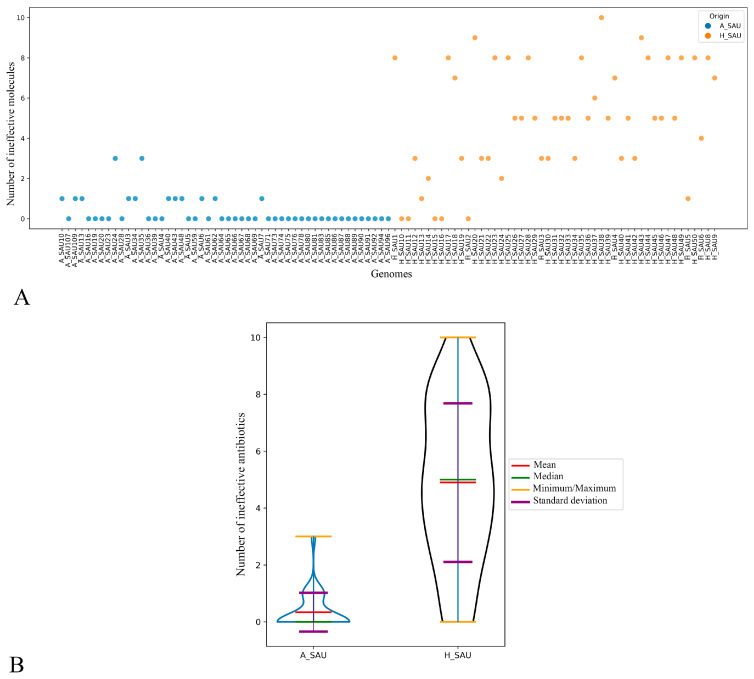
Comparative Burden of Ineffective Antibiotics. Scatter plot (**A**) and violin plot (**B**). The scatter plot (**A**) illustrates the number of antibiotics each genome is resistant to, grouped by origin (blue animal strains, orange human isolates). The violin plot (**B**) represents the distribution of the number of resistances for each group. Each “violin” represents the frequency distribution of the values (resistance to antibiotics) observed in the two groups. Within each violin, the mean (red line), median (green line), range (yellow lines for minimum and maximum) and standard deviation (purple line) are indicated.

In the scatter plot (Figure 4A), most of the animal samples did not show any resistance or exhibit resistance to a maximum of two antibiotics. The plot shows a clear separation between animal- and human-derived strains, with animal isolates (n = 50) exhibiting a very low resistance burden (mean 0.34 resistant antibiotics per strain; median 0; range 0–3) and human isolates (n = 50) displaying a substantially higher load (mean 5 resistant antibiotics per strain; median 5; range 0–10). This pattern indicates that multiple resistance phenotypes are largely confined to the human population, where individual isolates frequently combine resistance to several classes, including beta-lactams (methicillin and penicillin), aminoglycosides (amikacin, gentamicin, tobramycin, kanamycin), macrolides and lincosamides (erythromycin, clindamycin), fluoroquinolones (ciprofloxacin), and occasionally rifampicin, mupirocin or tetracycline, whereas animal isolates are typically fully susceptible or resistant only to penicillin, with sporadic resistance to tetracycline. No animal isolates can be defined as MRSA.

The violin plot (Figure 4B) represents the distribution of the number of resistances for each group. Animal strains show a highly compressed distribution, with a median of 0 resistant antibiotics per isolate (interquartile range [IQR] 0–0; range 0–3), whereas human strains exhibit a much broader and right-shifted distribution, with a median of 5 resistances per isolate (IQR 2–7; range 0–10). This difference in distributions is statistically significant (e.g., Welch’s *t*-test or a non-parametric alternative; *p* < 0.0001), reflecting both the higher central tendency and the greater dispersion of resistance counts among human isolates compared with the tightly clustered, mostly fully susceptible profiles observed in animal isolates.

**Figure 5:**
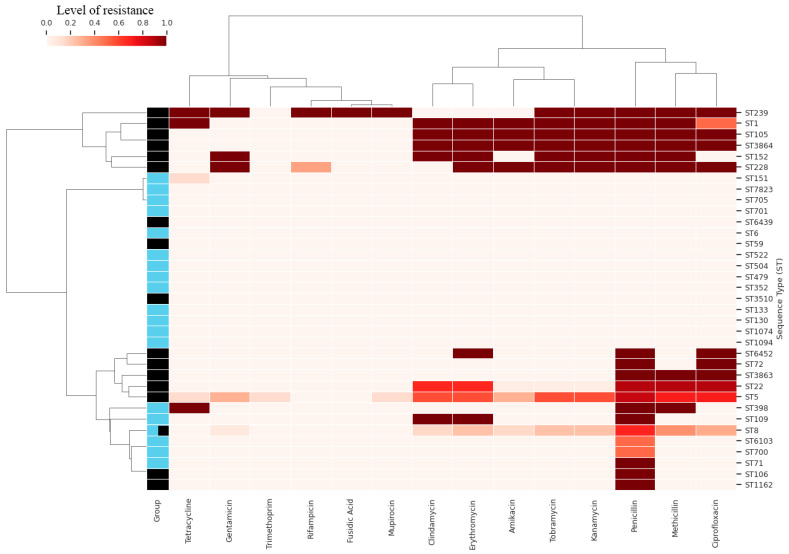
Heatmap and Hierarchical Clustering of Antimicrobial Resistance Profiles. Hierarchical clustering and heatmap analysis highlighting the level of resistance to multiple antibiotics across different *S. aureus* STs. Shades indicate quantitative resistance, revealing group-specific patterns and multidrug-resistant clones between animal derived strains (blue) and human derived isolates (black).

The heatmap (Figure 5) clusters the STs of the two groups based on the level of antibiotic resistance. Some STs assigned to the A_SAU group show no resistance, while most of the human samples are resistant to multiple antibiotics (as shown in the previous figures). The only ST common to the two groups is ST8, which however shows different levels of resistance between the two groups. Indeed, ST8 from animal isolates carried resistance determinants that were not detected in the human ST8 isolates, specifically *PC1_blaZ*, *AAC6_Ie_APH2_Ia*, and e*rmC.* Conversely, resistance genes identified exclusively in human ST8 isolates were *tet(K)*, *aad(6)*, *SAT-4*, and *APH(3′)-IIIa*. These differences may be useful to identify the source of the ST8 isolates (e.g., in an outbreak epidemiological investigation).

**Figure 6:**
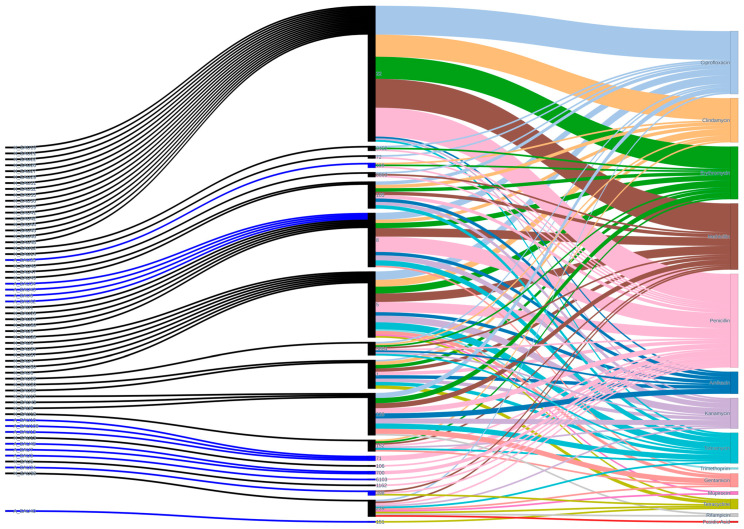
Sankey diagram illustrates the relationships between *S. aureus* isolates (**left**), sequence types (**center**), and their corresponding antibiotic resistance phenotypes (**right**). Animal-derived isolates are depicted in blue and human-derived isolates in black. The width of each ribbon is proportional to the number of isolates sharing a specific sequence type or resistance profile. This visualization reveals the diversity and overlaps of resistance patterns across host origins and facilitates the identification of multidrug-resistant lineages prevalent in both animal and human populations.

Among the antibiotics analyzed, penicillin is the one towards which most STs show resistance, followed by methicillin, ciprofloxacin, erythromycin and clindamycin (Figure 6).

**Figure 7:**
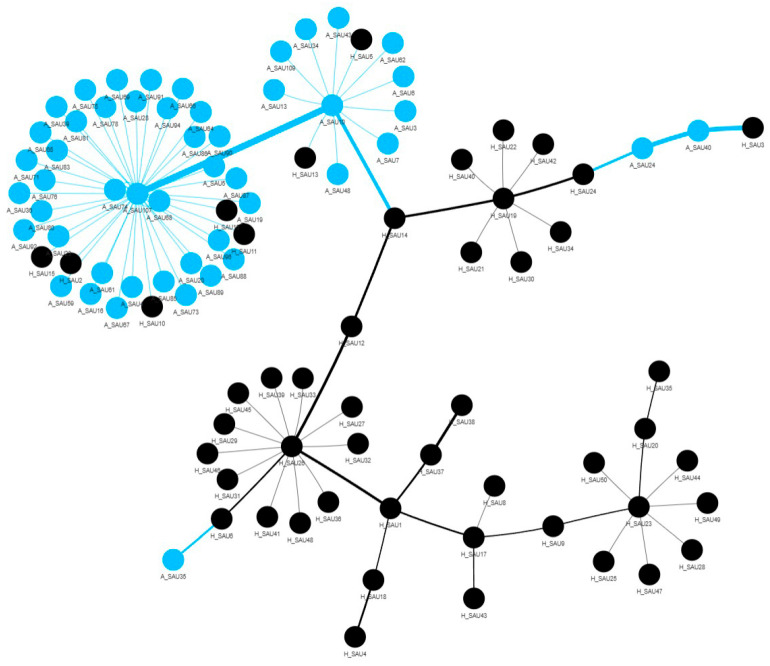
Minimum spanning network showing the genetic distance and relatedness among *S. aureus* isolates. Node colors distinguish human-(black) and animal-(blue) derived genomes, with clusters and bridges reflecting transmission or genetic divergence events.

The Minimum Spanning Tree (MST, Figure 7) highlights clusters of related strains based on the number of antibiotic resistances genes (ARGs). Two main clusters can be observed: one in the top left, composed almost exclusively of animal samples, and one in the bottom, predominantly human. The human cluster appears more fragmented, with several sub-clusters. This could indicate a greater diversity of genetic resistance profiles in the human STs, perhaps due to different selective pressure. The presence of nodes of opposite color in the two clusters (A_ or H_), suggest that STs that share similar resistance profiles in both animal and human samples. These nodes are particularly interesting because they could represent interspecific transmission events or the emergence of clones adapted to both hosts.

**Figure 8:**
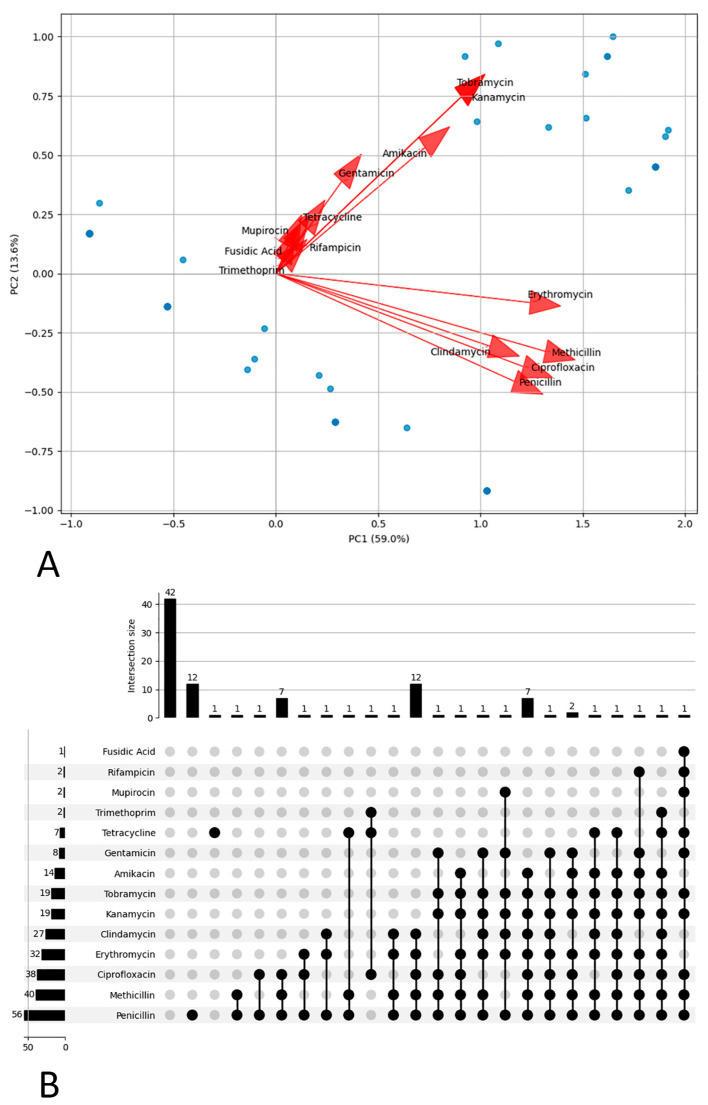
Distribution of Antimicrobial Resistance. (**A**) Principal component biplot depicting variation in resistance phenotypes across isolates, with antibiotic vectors indicating discriminatory power. (**B**) UpSet plot summarizing the intersection size for resistance to different antibiotics.

The Principal Component Analysis (PCA) results (Figure 8A) show that the two principal components together explain approximately 72.64% of the total variance in the resistance gene data, with PC1 alone accounting for 59% and PC2 for 13.64%. The PC1 principal component represents the main axis of variation, while PC2 adds a complementary dimension. Analyzing the contributions of the ARGs to the principal components, it is observed that PC1 is characterized by methicillin (0.439), erythromycin (0.414), ciprofloxacin (0.403), clindamycin (0.349), penicillin (0.389) and kanamycin/tobramycin (approximately 0.30) ARGs. These results indicate that variability in resistance to these antibiotics is the main factor distinguishing the strains along PC1. PC2, with positive contributions from kanamycin, tobramycin, amikacin and gentamicin ARGs, may reflect a secondary dimension of variation.

The analysis of ARGs combinations by means of the UpSet plot (Figure 8B) allows to monitor the evolution of resistance profiles and to detect the appearance of new multiresistance patterns. The results show that the most frequent combinations involve several ARGs: amikacin, tobramycin, kanamycin, methicillin, penicillin, clindamycin, erythromycin and ciprofloxacin. The recurrence of these combinations in many strains suggests the presence of co-selected resistance mechanisms. Aminoglycosides (amikacin, tobramycin and kanamycin) ARGs are frequently associated, which is consistent with the presence of aminoglycoside-modifying enzymes (AMEs) that confer cross-resistance to multiple molecules of this class. The concomitant presence of resistance to beta-lactams (methicillin and penicillin) indicates the spread of strains by multiple mechanisms, such as beta-lactamase production or alterations of the PBP (penicillin-binding protein) target.

Increased resistance in human-derived isolates, particularly to first-line agents such as penicillin, methicillin, erythromycin, clindamycin and ciprofloxacin, has direct implications for empirical therapy and the risk of treatment failure in clinical settings. The lower resistance burden in bovine isolates indicates that dairy herds may act as reservoirs of resistant *S. aureus*, with potential spillover into humans through occupational exposure or the food chain, reinforcing the need for coordinated antimicrobial stewardship under a One Health framework.

### 2.5. Resistance Genes and Virulence Factors

**Figure 9:**
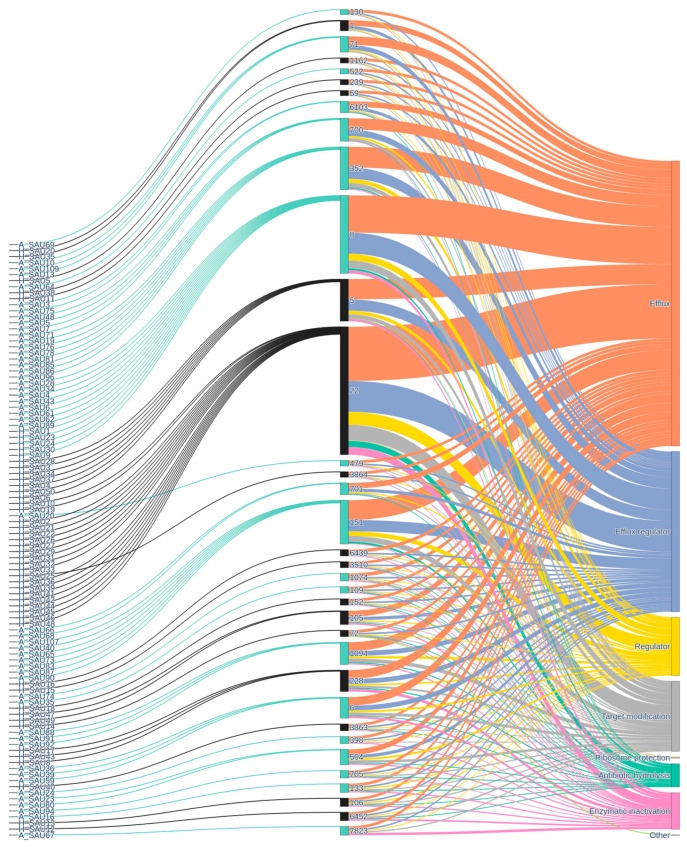
Sankey diagram traces the flow from *S. aureus* isolates (**left**), segregated by animal (light green) and human (black) origin, through sequence type (**center**), to specific antibiotic resistance genes (**right**). Colored link highlight connections between host source, genetic background, and resistance, offering a comprehensive view of the spread and diversity of antimicrobial resistance within and between host reservoirs, and identifying linkages between sequence types and multidrug-resistant profiles.

Figure 9 shows the relationships among the bacterial strains analyzed, their STs, the ARGs identified, and the associated molecular mechanisms. The first level, consisting of the strains, shows significant diversity, with numerous isolates distributed across different STs, suggesting considerable genetic heterogeneity and the possible presence of clones. The strain → ST transition highlights some particularly well-represented STs, which act as hubs in the network (i.e., ST22, ST8, ST352, ST151). This pattern may reflect the presence of clones that have spread across different contexts, probably due to selective advantages conferred by resistance or virulence. Some STs are associated with a broad spectrum of ARGs, while others show a more limited carriage (ST7823). The presence of multiple ARGs in some STs suggests the co-localization of mobile genetic elements, such as plasmids or transposons, which facilitate their horizontal spread. The last level, resistance gene → mechanism, represents the functional categorization of the identified genes. Here, some dominant mechanisms clearly emerge, such as enzymatic modification of the antibiotic, target modification, active efflux and target protection.

**Figure 10:**
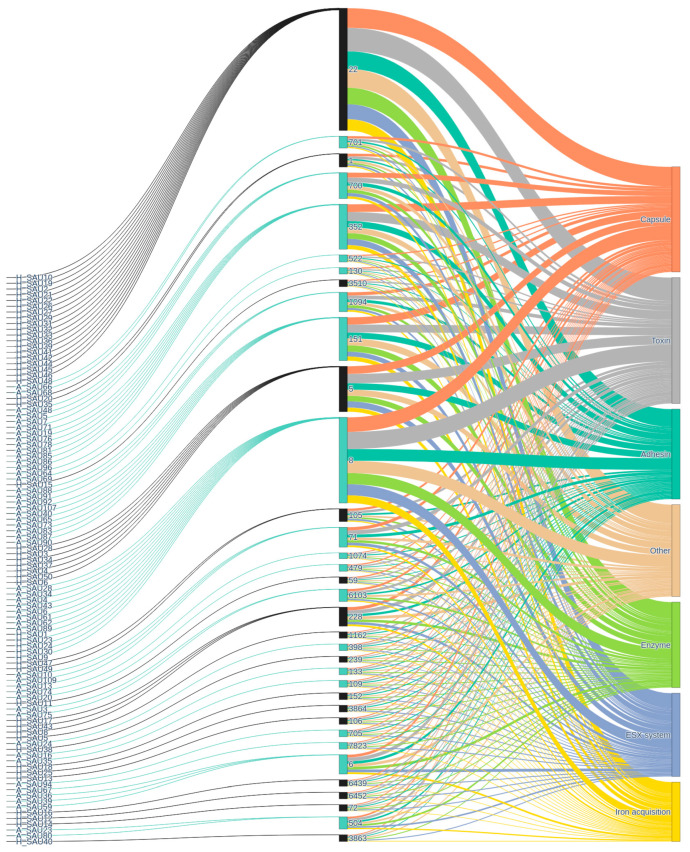
Distribution of virulence factors across *S. aureus* ST and functional classes. Sankey diagram illustrating the flow from *S. aureus* isolates, divided in animal (blue) and human (black) origin (**left**), through ST (**center**), to categorized virulence factor classes (**right**). This visualization highlights the diverse distribution of virulence genes related to capsule formation, toxins, adhesins, enzymes, secretion systems, iron acquisition, and other functions.

Figure 10 shows the distribution and association of virulence factors in the strains analyzed. Starting from the isolates, the graph shows a structure similar to that observed for resistance, with numerous samples converging on a relatively limited number of STs. This pattern suggests that, even for virulence, there are dominant clones that serve as the main carriers of certain virulence factors within the population. The strain → ST transition again highlights some ST, which bring together numerous strains (ST22, ST8), suggesting a complex clonal structure and the possible presence of hypervirulent clones. Some STs are associated with a broad spectrum of virulence factors, which may facilitate adaptation to different hosts or ecological niches. Other STs, on the other hand, show a “specialization” towards certain categories (penicillin and tetracycline ARGs), indicating a possible narrower ecological niche or infection strategy (ST109, ST152, ST106). The presence of numerous connections between STs and virulence categories suggests that genetic plasticity affects not only resistance but also pathogenic potential. The figure shows that some categories of virulence factors are particularly well represented, such as adhesins and toxins, while others, such as immune evasion factors or degradative enzymes, are less frequent or more specific to certain STs.

**Figure 11:**
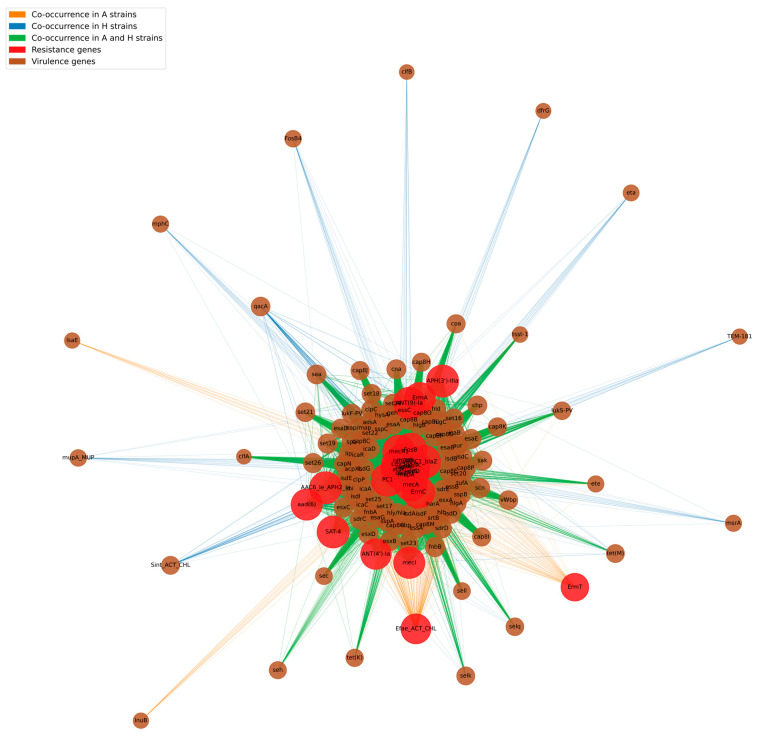
Network of resistance and virulence gene co-occurrence in *S. aureus*. Network graph depicts the co-occurrence patterns of antimicrobial resistance (red nodes) and virulence genes (brown nodes) in *S. aureus* isolates. Edge colors differentiate co-occurrence within animal strains (orange), human strains (blue), or both (green), as indicated in the legend. Node size is proportional to gene connectivity, highlighting core resistance determinants and the diversity of accessory virulence factors across host backgrounds. This representation provides insight into the potential for co-selection and dissemination of clinically relevant gene combinations.

To better determine the relationship between resistance and virulence genes, a network analysis was performed focusing on co-occurrence (Figure 11). The arcs connecting the nodes are colored orange if the co-occurrence is exclusive to the A_ strains, purple if exclusive to the H_ strains, and lilac if both groups share the co-occurrence. The size of the nodes is proportional to the degree of connection (i.e., the number of genes with which each resistance or virulence gene co-occurs), while the thickness of the arcs reflects the frequency of co-occurrence. The network is characterized by most resistance genes connected to many virulence factors, and vice versa. This suggests that, in the strains analyzed, resistance genes are not isolated events but are accompanied by several virulence genes, highlighting the genetic plasticity of *S. aureus.* Most co-occurrences are shared between the two groups of strains (lilac), while only a minority of arcs are exclusive to strains A_ (orange) or H_ (purple). This suggests that the structure of the co-occurrence network is largely conserved between the two groups.

## 3. Discussion

*Staphylococcus aureus* has emerged as a paradigm of host adaptation, clonal evolution, and global distribution among pathogens with zoonotic and anthropozoonotic potential. The results of our comparative genomic analysis of staphylococcal strains isolated from milk samples and human clinical sources expand current knowledge on host-specialized lineages, genetic diversification, and the mechanisms underlying host jumps. We critically discuss the implications of these models, referring to comparative genomics, population-level investigations, phylogenetics, antimicrobial resistance (AMR), and virulence traits.

The clonal diversity of *S. aureus* in both humans and cattle reflects a global trend that determines the epidemiology in each host. Our data, in line with other investigations, confirm that CC97, CC133, and CC151 are the main complexes adapted to cattle, consistent with the results of molecular and genomic typing studies demonstrating their prevalence in dairy herds in Europe, North America, Asia, and Africa [1,28,29]. This is reflected in human populations by dominant clonal complexes such as CC1, CC5, CC8, CC30, and CC45 [29,30]. Several studies report the presence of “human” lineages (particularly CC8 and CC5) in cattle and, conversely, the detection of “zootechnical” lineages such as ST398 in human carriers, especially among individuals who work closely with livestock [29,31,32,33]. Our results support these findings, with evidence of sporadic host shifts and complex patterns of clonal overlap, although true sustained host adaptation appears relatively rare for most lineages. Bovine-adapted lineages are characterized by the acquisition of specific phages and pathogenicity traits, supporting genetic events that facilitate adaptation to the bovine mammary niche. Human-adapted strains often contain prophages that modify β-hemolysin and encode immune evasion factors particular to humans [34]. It is crucial to acknowledge that transitions from human to bovine, exemplified by CC8, involve the loss of human-specific phage elements concomitant with the acquisition of novel SCC cassettes, occasionally encoding proteins featuring the new LPXTG motif. This phenomenon appears to facilitate rapid adaptation, as evidenced by our research and by comparative microarray-based genomics conducted in China and Switzerland, respectively [32,33,35].

Principal component analysis (PCA), and minimal spanning tree (MST) are examples of multivariate statistical studies that help us understand the population structure. PCA shows that resistance to important clinical antibiotics like methicillin, erythromycin, and ciprofloxacin is the main factor that separates strains. Resistance to macrolides and lincosamides (erythromycin and clindamycin) is prevalent in the most common combinations, probably mediated by ribosomal target modifications or efflux pumps. Co-resistance of beta-lactams and aminoglycosides is frequently associated with plasmids with multiple gene cassettes, while resistance to macrolides and lincosamides may arise from *erm* or *mef* genes, often co-localized with other resistance genes. Moreover, the influence of beta-lactam antibiotics (methicillin and penicillin) and macrolides (erythromycin and clindamycin) ARGs on PC1 underlines the importance of closely monitoring resistance to these classes, which represent the first choice for empirical therapy. In addition, the relevant contribution of ciprofloxacin highlights the need to also consider resistance to fluoroquinolones [36,37]. The MST shows distinct clusters that are mostly based on the host’s origin, but there are also important bridging nodes that signal genetic overlaps and possibly cross-transmission or shared environmental reservoirs. This supports the idea that pathogens’ evolution and ecology are connected in ways that make it harder to develop controls measures to manage them separately. Therefore, we need to think about diversity in pathogen genomics in terms of ecology and evolution.

Recent studies that used global phylogenetic reconstruction show that clonal complexes switch hosts at different rates and in different ways. The dairy bovine population acts more like a “sink” than a “source” for host hops, save for certain lineages like CC425 [38]. Modeling shows that ecological factors including farming practices, antibiotic use, and herd management, as well as microevolutionary adaptation, all affect how populations of cattle and people change over time [38,39,40]. In our strains, the presence of compact clades consisting exclusively of animal or human strains suggests that, despite the possibility of transmission between species, there are still selective or ecological barriers that favor the specialization of certain evolutionary lines. The presence of mixed clades, on the other hand, suggests that in some circumstances these barriers can be overcome, allowing the transmission of strains between animals and humans.

*S. aureus* can swap hosts and spread from animals to people because it has a lot of genetic variation and can adapt to new environments. Finding different but overlapping clonal populations shows that some strains may be better suited to certain hosts, but ecological barriers are not always strong enough to stop genetic exchange and adaptation. This ability to adapt is made worse by the continual antibiotic selective pressures that cause multi-drug resistance phenotypes to appear, especially in the clinical setting for humans. The fact that animal strains have a lower but still significant level of resistance means that there is still a risk of reservoirs existing in livestock populations, which could be sources of resistant diseases that could spread to people. Resistance genes such as *tet(38)*, *dfrC*, *norA*, *norC*, *sepA*, *mepA*, *mgrA*, *sdrM*, *arlS*, *arlR*, *mepR*, *kdpD*, *LmrS* are associated with a wide range of virulence factors (*set22*, *cap8G*, *fnbB*, *clpC*, *hly/hla*, *ebp*, *esaD*, *sspA*, *icaR*, etc.). This breadth of co-occurrences suggests that most strains possess multiple resistance and virulence determinants simultaneously. The central structure of the network, in which resistance nodes are most represented (such as *tet(38)*, *norA*, *norC*, *dfrC*, *sepA*, *mepA*, *mgrA*), shows a high degree of connection, indicating that these genes play a key role in the bacterial population analyzed. They could represent “core” genes, whose presence is almost ubiquitous, and which act as a platform for the acquisition or co-localization of additional virulence genes. Because of the complicated genetic networks and the fact that resistance and virulence genes often happen at the same time, interventions that only look at resistance may miss important parts of how pathogens spread and how they cause disease.

However, this study has some limitations that should be considered when interpreting the results. First, the sample size and restriction to dairy herds from a single geographic region limit the generalizability of the findings to other production systems and epidemiological contexts. Second, human comparator genomes were selected from Italian public database entries and lack detailed and harmonized metadata on clinical presentation, which constrains inferences on directionality and frequency of cross-species transmission. Finally, the analysis is based on genomic prediction of resistance and virulence rather than systematic phenotypic characterization, so functional consequences of specific gene constellations should be confirmed in future experimental and longitudinal studies.

These results show how important it is to have integrated surveillance systems that can follow the evolution of resistance in *S. aureus* and the ways it spreads across both human and animal populations. This supports the One Health concept for addressing antibiotic resistance and zoonotic diseases caused by *S. aureus*. Reducing AMR requires coordinated efforts of human health care, veterinary medicine, farming, and environmental management. To stop the emergence of resistant and virulent *S. aureus* strains, it is important to use antibiotics wisely in both clinical and agricultural contexts, improve genomic surveillance, and take targeted steps to prevent infections. Further studies on the molecular mechanisms that allow hosts to adapt and pathogens to become resistant could aid in the development of new interventions to reduce the spread of this pathogen.

## 4. Materials and Methods

### 4.1. Bacterial Isolation and Identification

Isolates were collected in the period January 2024–May 2025 from the dairy herds in Lombardy region applying a mastitis control program and delivering samples to ARAL (Associazione Regionale Allevatori Lombardia) laboratories. Only *S. aureus* isolated from quarter milk samples of cows with subclinical mastitis were considered. Diagnosis was performed following current diagnostic protocols applied in ARAL laboratories and following standardized procedures [4,41]. A positive *S. aureus* mastitis was defined when the bacteria was found in pure culture or when no more than two different bacteria species were observed. For each herd, a single isolate was considered. When more than one cow was positive within a herd, the selection criteria were: presence of a pure culture and highest somatic cell count.

The bacteriological analysis of the single-quarter milk samples was conducted in accordance with the guidelines established by the National Mastitis Council (NMC). Milk samples were inoculated onto Tryptic Soy Agar (TSA, Microbiol, Uta, Cagliari, Italy) supplemented with 5% defibrinated sheep blood, and Baird-Parker agar plates (Thermo Fisher, Milan, Italy), subsequently incubated aerobically at 37 °C for 24 h. Colonies presumptively identified as staphylococci based on golden-yellow pigmentation and hemolytic activity, underwent Gram staining. After staining, confirmatory tests include catalase, coagulase and biochemical tests performed on Vitek^®^ system (BioMerieux, Lyon, France).

### 4.2. DNA Extraction

Isolated colonies from fresh overnight culture of *S. aureus* on TSA agar were resuspended in 500 μL of PBS (Phosphate-Buffered Saline, Euroclone, Milan, Italy) and centrifuged at 10,000× *g* for 1 min at room temperature. DNA was extracted using the Quick-DNA™ HMW MagBead kit (Zymo Research, Irvine, CA, USA) in accordance with the manufacturer’s guidelines.

The quantity and quality of the extracted DNA were evaluated using UV-Vis spectrophotometry with the NanoReady Touch Micro Volume Reader (Aurogene, Rome, Italy), confirming that the A_260_/A_280_ and A_260_/A_230_ ratios fell within the range of 1.8 to 2, respectively.

### 4.3. Sequencing and Bioinformatics Analyses

Genomic libraries were constructed using 200 ng of input DNA per sample, which underwent tagmentation using transposase with the Rapid Barcoding Sequencing kit (SQK-RBK114.96, Oxford Nanopore Technologies, Oxford, UK). Subsequently, twelve strains were loaded on a single flowcell (FLO-MIN114, version R10.4.1) and sequenced using the third-generation MinION Mk1C sequencer (Oxford Nanopore Technologies, Oxford, UK) for up to 72 h.

Dorado (v0.8.2, [42]) was used for basecalling, adapter trimming, and demultiplexing. Summary data about the quality and amount of reads were generated using NanoPlot (v1.44.0, [43]). Filtration was conducted using FiltLong (version 0.2.1, [44]). Subsequently, the genomes were de novo assembled using Flye (v2.8.1-b1676, [45]), and the resulting contigs were polished and corrected via Medaka (v2.0.1, [46]). The assessment of genomic completeness and contamination was conducted using CheckM2 (v1.2.4, [47]). Structural and functional genome annotation was performed using the NCBI Prokaryotic Genome Annotation Pipeline (PGAP, [48]), which identifies and annotates coding sequences, rRNAs, and tRNAs. Pan-genome analyses, including identification of core and accessory genes and construction of the pangenome matrix, were carried out with PGAP2 [49] and complementary tools such as Anvi’o [50]. Average nucleotide identity was assessed using ANIClustermap tool [51] and the phylogenetic tree was visualized with iTOL (v.6) [52].

The genomes of the isolated bacteria were compared with those in the database (Pathogen Detection, GenBank, https://www.ncbi.nlm.nih.gov/pathogens/ accessed on December 2024) to analyze pathogenic features, including clonality and the presence of antibiotic resistance genes. Fifty genomes of *S. aureus*, obtained from human isolates, were randomly chosen from samples collected in Italy (Supplemental Table S1).

### 4.4. Statistical Analysis

A Chi-squared test was used for categorical variables (e.g., resistant vs. susceptible strains, animal vs. human), and Welch’s *t*-test for continuous variables (e.g., number of resistance or virulence genes per isolate); a *p*-value < 0.05 was deemed statistically significant for all analyses. Analyses (Chi-squared test and inspection of variable distributions by histograms, Q–Q plots, and boxplots prior to parametric testing) were originally performed in Excel™ (Microsoft Corp., Redmond, WA, USA).

Principal component analysis (PCA) was performed on the antibiotic resistance profiles using scikit-learn’s PCA implementation in Python (v3.13), retaining two principal components. Each isolate was represented by its numeric resistance values across antibiotics; features were centered (default behavior of PCA in scikit-learn) but not scaled to unit variance, and principal components were obtained from the eigendecomposition of the covariance matrix, with loadings computed as component coefficients multiplied by the square root of the explained variance and visualized in a biplot.

For clustering, pairwise dissimilarities between isolates were computed on binary (0/1) resistance profiles using the Jaccard distance, and agglomerative hierarchical clustering with average linkage was applied to generate a dendrogram of isolates. The same Jaccard distance matrix was used to construct a complete weighted graph in Python (Linux environment), from which a minimum spanning tree (MST) was obtained using a standard minimum-spanning-tree algorithm and visualized with node colors indicating isolate origin.

## 5. Conclusions

The results of this study are broadly consistent with current knowledge on *Staphylococcus aureus*, while providing additional resolution on host association and genomic diversity. The data indicate strong, although not absolute, host adaptation, together with a dynamic capacity for host switching mediated by mobile genetic elements (MGEs) and patterns of antimicrobial resistance. The analyses further identify lineage-specific adaptation events and reveal complex transmission pathways involving both animal and human populations, underscoring the importance of integrated, genomics-informed surveillance.

Despite these advances, important gaps remain in understanding the relationship between genomic variation and pathogenic potential. In particular, the mechanisms that constrain or facilitate host jumps, as well as the ecological determinants that shape local and global distribution patterns, are still only partially resolved. Addressing these gaps will require a comprehensive approach that integrates comparative genomics, population-level epidemiology, experimental studies of pathogenesis, and multi-host ecological investigations. Such an integrated framework is essential to improve the control of a pathogen that exhibits adaptable and dynamic host interactions in both bovine and human populations, and to inform coordinated monitoring systems that are globally connected, genomics-based, and explicitly ecology-aware.

## Figures and Tables

**Table 1:** Characteristics and accession numbers of the 50 *S. aureus* strains sequenced.

Sample Name	Genome	Biosample	Comp ^a^	Cont ^b^	CDS ^c^	Cov ^d^	Genome Length (Mbp)	Contigs	GC (%)	ST	Agr_Type
A_SAU10	JBRUVB000000000	SAMN47618783	100.0	0.07	2474	135	2.71	6	32.9	151	gp1
A_SAU107	CP186908	SAMN47618826	100.0	0.16	2571	259	2.73	1	32.8	71	gp2
A_SAU109	CP186909	SAMN47618827	100.0	0.05	2511	322	2.74	1	32.8	71	gp1
A_SAU13	JBRUVC000000000	SAMN47618784	100.0	0.09	2506	290	2.74	2	32.9	71	gp1
A_SAU16	JBRUVD000000000	SAMN47618785	100.0	0.17	2575	404	2.79	2	33.2	133	gp1
A_SAU19	JBRUVE000000000	SAMN47618786	100.0	0.08	2556	471	2.76	2	32.9	352	gp1
A_SAU20	CP186883	SAMN47618787	99.98	0.47	2682	347	2.78	1	32.8	479	gp2
A_SAU23	CP186884	SAMN47618788	100.0	0.21	2509	100	2.68	1	32.8	504	gp2
A_SAU24 ^e^	JBRUVF000000000	SAMN47618789	100.0	0.15	2555	361	2.8	2	33.0	398	gp1
A_SAU28	CP186885	SAMN47618790	100.0	0.12	2545	73	2.79	1	32.8	8	gp1
A_SAU3	JBRUUX000000000	SAMN47618778	100.0	0.12	2535	209	2.79	2	32.7	8	gp1
A_SAU34	JBRUVG000000000	SAMN47618791	100.0	0.13	2584	91	2.82	2	32.7	109	gp1
A_SAU35	CP186886	SAMN47618792	100.0	0.2	2730	249	2.89	1	32.8	6	gp2
A_SAU36	JBRUVH000000000	SAMN47618793	100.0	0.11	2422	363	2.7	2	32.9	6	gp1
A_SAU39	CP186887	SAMN47618794	100.0	0.1	2415	116	2.69	1	32.9	6103	gp1
A_SAU4	CP186882	SAMN47618779	100.0	0.08	2533	146	2.77	1	32.8	151	gp1
A_SAU40	JBRUVI000000000	SAMN47618795	100.0	0.69	2641	40	2.77	2	32.8	8	gp2
A_SAU43	JBRUVJ000000000	SAMN47618796	100.0	0.13	2599	418	2.84	2	32.7	700	gp1
A_SAU48	JBRUVK000000000	SAMN47618797	100.0	0.64	2709	380	2.88	2	32.9	8	gp3
A_SAU5	JBRUUY000000000	SAMN47618780	100.0	0.22	2684	166	2.88	2	33.4	6	gp3
A_SAU59	CP186888	SAMN47618798	100.0	0.1	2482	290	2.74	1	32.9	700	gp1
A_SAU6	JBRUUZ000000000	SAMN47618781	100.0	0.12	2534	165	2.79	2	32.8	8	gp1
A_SAU61	JBRUVL000000000	SAMN47618799	100.0	0.13	2588	240	2.82	2	32.7	8	gp1
A_SAU62	JBRUVM000000000	SAMN47618800	100.0	0.12	2532	116	2.79	2	32.8	522	gp1
A_SAU64	CP186889	SAMN47618801	100.0	0.38	2564	202	2.78	1	32.9	151	gp1
A_SAU65	CP186890	SAMN47618802	100.0	0.29	2617	265	2.77	1	32.8	701	gp2
A_SAU66	CP186891	SAMN47618803	100.0	0.22	2587	197	2.79	1	32.9	7823	gp1
A_SAU67	CP186892	SAMN47618804	100.0	0.08	2481	212	2.74	1	32.8	701	gp1
A_SAU68	CP186893	SAMN47618805	100.0	0.22	2586	203	2.74	1	32.9	130	gp1
A_SAU69	CP186894	SAMN47618806	100.0	0.26	2524	294	2.78	1	32.9	8	gp3
A_SAU7	JBRUVA000000000	SAMN47618782	100.0	0.53	2705	355	2.9	3	33.3	700	gp3
A_SAU71	CP186895	SAMN47618807	100.0	0.27	2660	326	2.84	1	32.9	151	gp3
A_SAU73	CP186896	SAMN47618808	100.0	0.18	2618	182	2.77	1	32.8	1074	gp2
A_SAU74	CP186897	SAMN47618809	100.0	0.18	2556	266	2.72	1	32.8	6103	gp2
A_SAU75	CP186898	SAMN47618810	100.0	0.26	2489	237	2.74	1	32.8	352	gp1
A_SAU76	JBRUVN000000000	SAMN47618811	100.0	0.08	2624	229	2.84	12	32.8	352	gp1
A_SAU78	JBRUVO000000000	SAMN47618812	100.0	0.08	2545	290	2.77	2	32.9	700	gp1
A_SAU80	CP186899	SAMN47618813	100.0	0.13	2590	181	2.74	1	32.8	504	gp2
A_SAU81	CP186900	SAMN47618814	100.0	0.06	2536	256	2.76	1	32.9	352	gp1
A_SAU83	CP186901	SAMN47618815	100.0	0.18	2513	309	2.69	1	32.8	151	gp2
A_SAU85	JBRUVP000000000	SAMN47618816	100.0	0.08	2558	118	2.78	2	32.9	352	gp1
A_SAU86	JBRUVQ000000000	SAMN47618817	100.0	0.08	2558	232	2.78	2	32.9	352	gp1
A_SAU87	CP186902	SAMN47618818	100.0	0.29	2615	182	2.77	1	32.8	151	gp2
A_SAU88	JBRUVR000000000	SAMN47618819	100.0	0.27	2828	201	2.98	4	32.9	1094	gp1
A_SAU89	CP186903	SAMN47618820	100.0	0.13	2559	260	2.8	1	32.8	8	gp1
A_SAU90	CP186904	SAMN47618821	100.0	0.17	2620	179	2.77	1	32.8	151	gp2
A_SAU91	JBRUVS000000000	SAMN47618822	100.0	0.27	2793	230	2.95	3	32.9	1094	gp1
A_SAU92	CP186905	SAMN47618823	100.0	0.29	2837	186	2.97	1	33.0	1094	gp1
A_SAU94	CP186906	SAMN47618824	100.0	0.19	2509	263	2.68	1	32.8	705	gp2
A_SAU96	CP186907	SAMN47618825	100.0	0.06	2544	315	2.76	1	32.9	352	gp1

^a^ Comp = Completeness; ^b^ Cont = Contamination; ^c^ CDS = Coding Sequences; ^d^ Cov = Coverage; ^e^ A_SAU24: this is the only isolate which was positive for *mecA* gene and clustered as SCCmec type V.

## Data Availability

All sequences were deposited in NCBI under the Bioproject PRJNA1243330.

## Supplementary Materials

The following supporting information can be downloaded at https://www.mdpi.com/article/10.3390/antibiotics15010098/s1. Table S1: Characteristics and accession numbers of the 50 H_ *S. aureus* strains. Figure S1: Preprocessing quality metrics and feature-level consistency of Animal-derived *Staphylococcus aureus* genomes. Figure S2: Pan-genome structure, gene cluster dynamics, and paralogy patterns across A_strains. Figure S3: Preprocessing quality metrics and feature composition of 50 H_ derived strains via PGAP2. Figure S4: Pan-genome architecture and gene cluster dynamics of 50 H_ *S. aureus* genomes analyzed with PGAP2.
