# Supplementary material for: Genomic Evidence Supporting a One Health Perspective on Staphylococcus aureus Bovine Mastitis
Source: Antibiotics (Basel). 2026 Jan 18;15(1):98. doi: 10.3390/antibiotics15010098 (PMC12837917; doi:10.3390/antibiotics15010098)

Supplemental figure S1: Preprocessing quality metrics and feature-level consistency of Animal-derived *Staphylococcus aureus* genomes. (A) Pairwise average nucleotide identity (ANI) values all above 96% indicate that all isolates belong to the same species and represent a genomically coherent dataset. (B) Distribution of the total number of predicted genes per genome (approximately 2,400–2,900 genes) shows limited variability in genome size and annotation depth across strains. (C) Counts of "half core" and "single cloud" features illustrate the balance between conserved and strain-specific gene families identified by the fine-grained feature network. (D) Proportions of genomes carrying each feature across the 50 strains demonstrate a continuous spectrum from core-like to rare accessory genes. (E) Start–stop codon usage profiles across genomes indicate consistent coding sequence boundary patterns with only minor variation among strains, further supporting annotation quality.

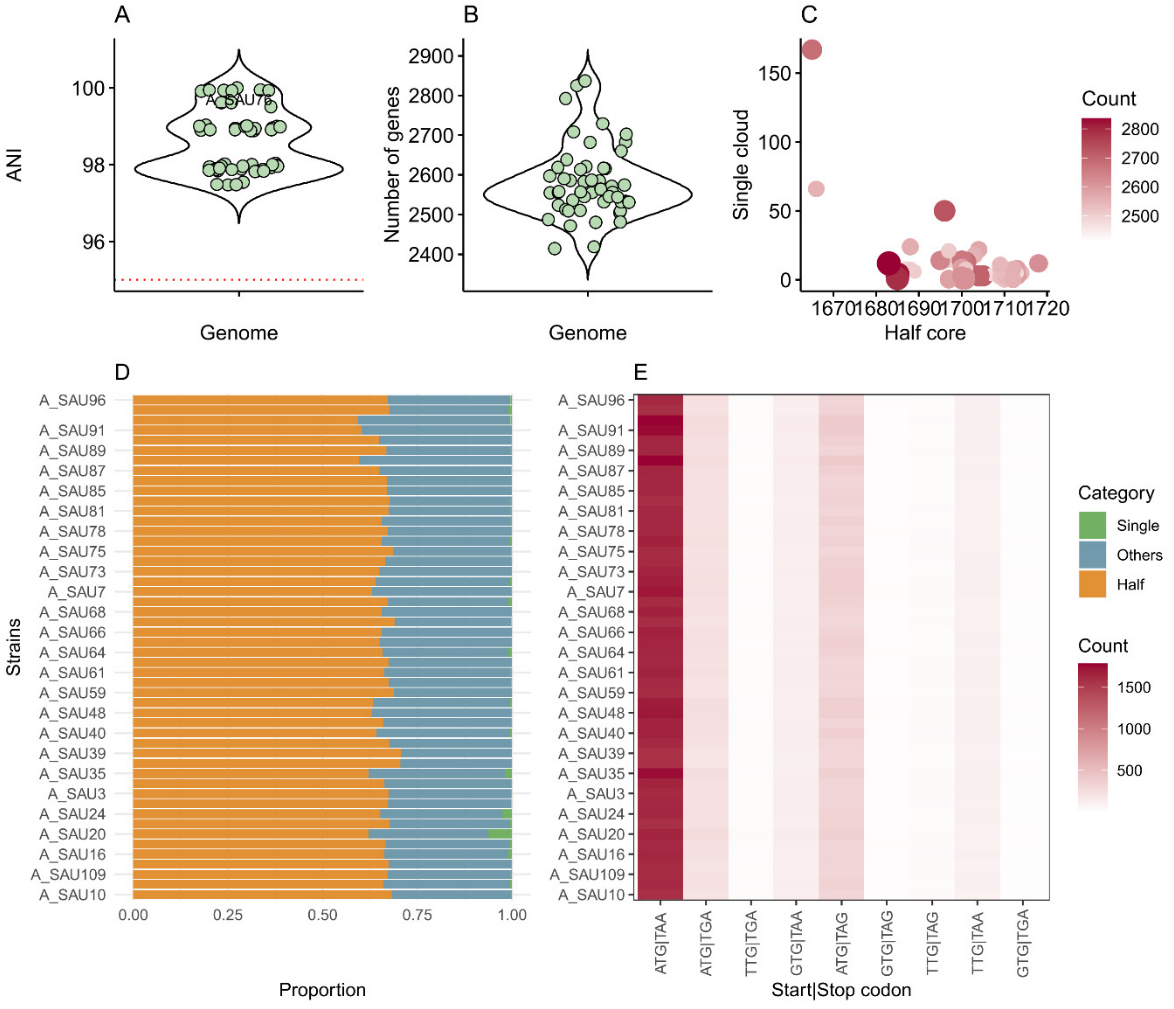

Supplement: Supplementary file 1 [file antibiotics-15-00098-s001.zip › Supplementary_S1.pdf]
