# Supplementary material for: Genomic Evidence Supporting a One Health Perspective on Staphylococcus aureus Bovine Mastitis
Source: Antibiotics (Basel). 2026 Jan 18;15(1):98. doi: 10.3390/antibiotics15010098 (PMC12837917; doi:10.3390/antibiotics15010098)
Supplement: Supplementary file 1 [file antibiotics-15-00098-s001.zip › Supplementary_S2.pdf]

Supplemental figure S2: Pan-Genome structure, gene cluster dynamics, and paralogy patterns across A_strains

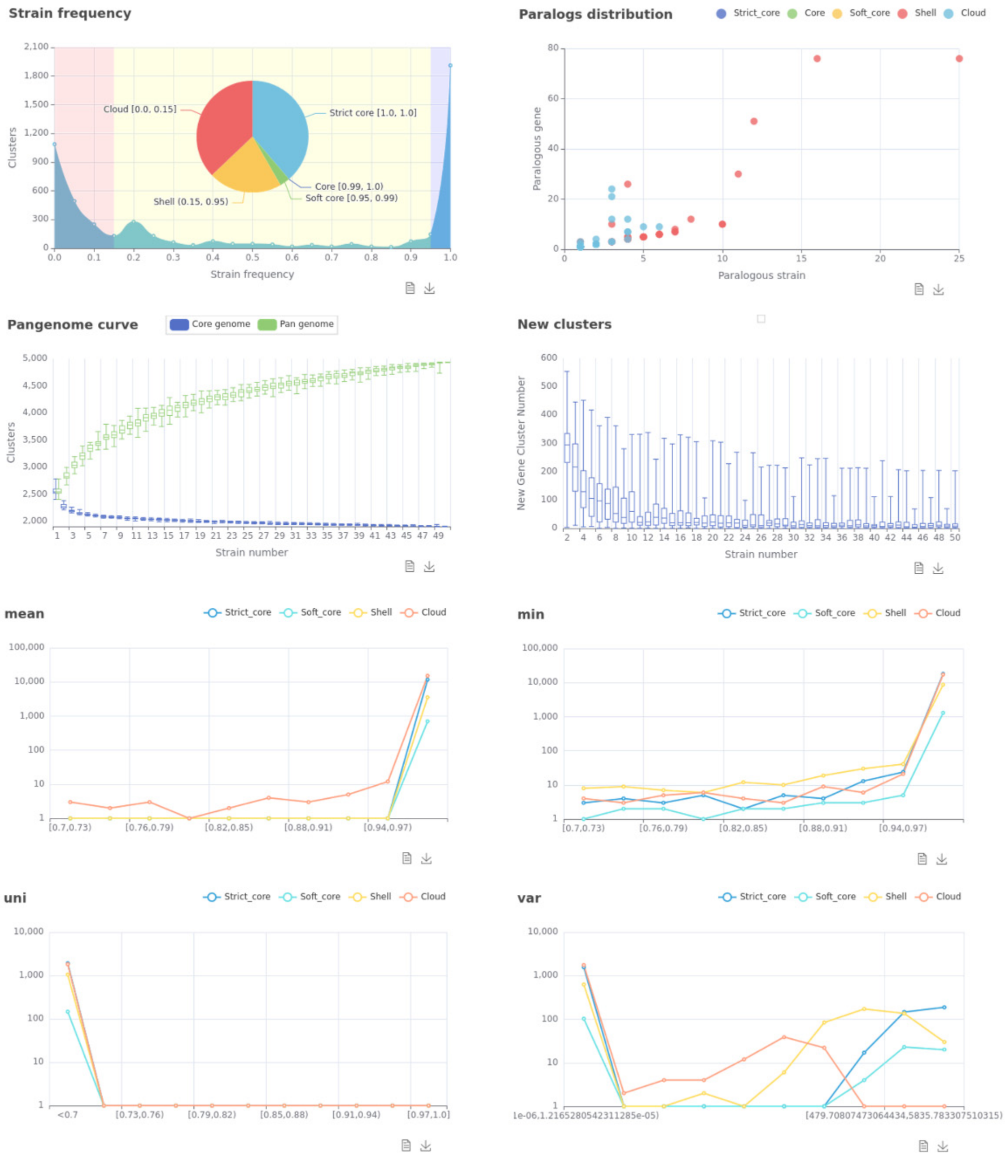

The post-processing panel shows that the 50 genomes have about a few hundred strict core clusters, about 1,000–1,500 additional core/soft-core clusters, and about the same or more shell and cloud clusters. Cloud genes are the most common at low frequencies (strain frequency ≤0.15). The pan-genome curve goes up from around 3,000–3,500 clusters in the first genomes to about 4,500–5,000 clusters when all 50 genomes are incorporated. At the same time, the core genome goes down from over 3,000 clusters to about 2,000–2,500 clusters and becomes closer to a plateau. The paralog distribution shows that a frequency class may have up to several dozen paralogous genes. Some strains even have paralogs for 10–20 clusters, which shows that there is a lot of duplication in both core/soft-core and shell/cloud compartments. The "new cluster" plots show that genomes added early on add hundreds of new gene clusters, while genomes added later add fewer and fewer (usually in the range of tens of new clusters per genome). These new clusters are mostly in the shell and cloud categories, which is consistent with diminishing returns but no clear saturation of pan-genome size.
