# Supplementary material for: Genomic Evidence Supporting a One Health Perspective on Staphylococcus aureus Bovine Mastitis
Source: Antibiotics (Basel). 2026 Jan 18;15(1):98. doi: 10.3390/antibiotics15010098 (PMC12837917; doi:10.3390/antibiotics15010098)

Supplemental figure S3: Preprocessing quality metrics and feature composition of 50 H_ derived strains via PGAP2. (A) Pairwise average nucleotide identity (ANI) values all over 96%, show that all isolates are from the same species and make up a genomically coherent dataset. (B) The total number of predicted genes per genome (around 2,400–2,800 genes) reveals that the sizes of genomes and the depth of their annotations don't change much across strains. (C) The number of "half core" and "single cloud" features shows the balance between gene families that are conserved and those that are particular to a certain strain, as shown by the fine-grained feature network. (D) The percentages of genomes with each characteristic among the 25 strains show a continuous range from core-like genes to uncommon accessory genes. (E) The use of start and end codons across genomes shows that coding sequence boundaries are the same in all strains, with only small differences.

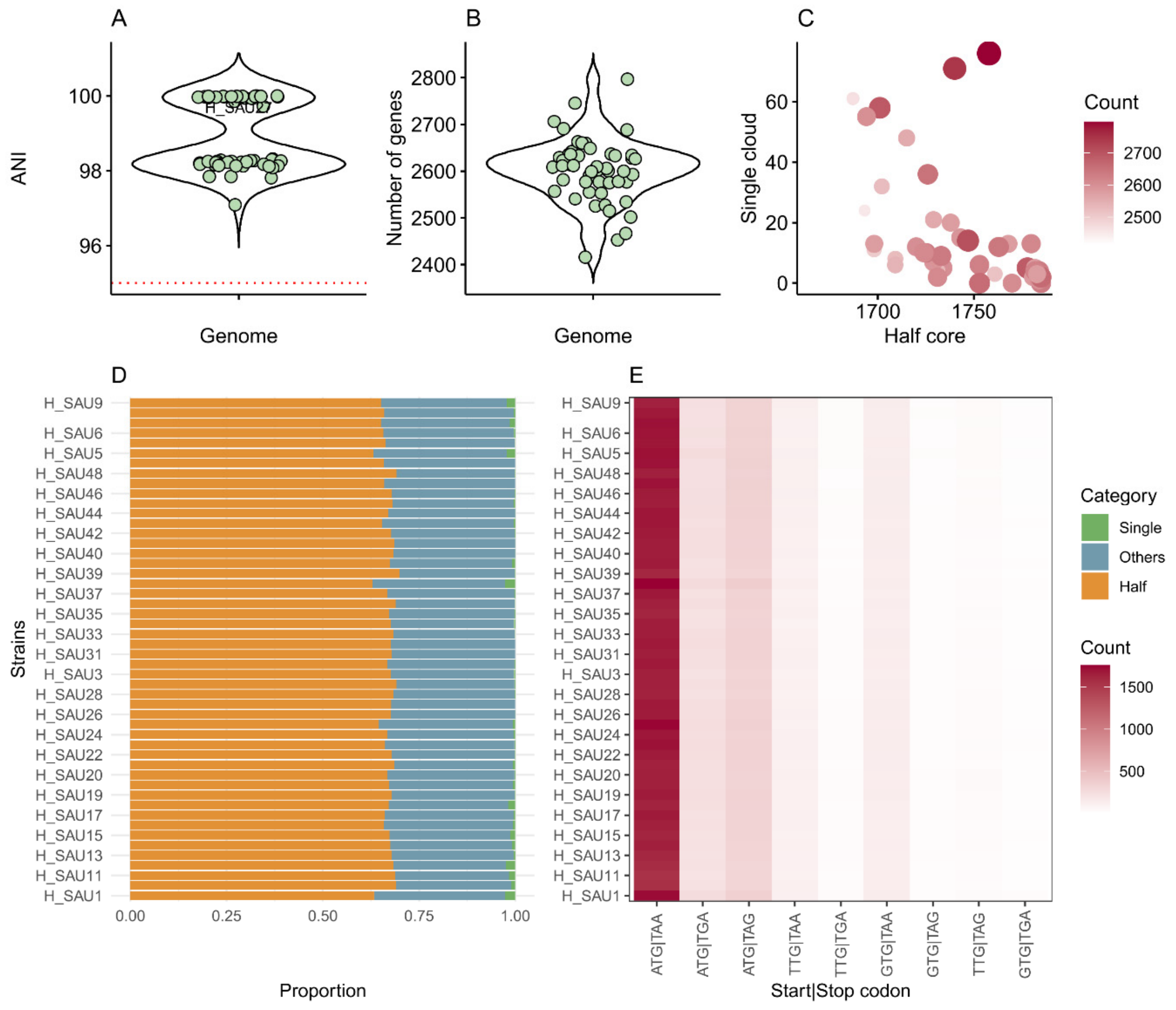

Supplement: Supplementary file 1 [file antibiotics-15-00098-s001.zip › Supplementary_S3.pdf]
