# Supplementary material for: Genomic Evidence Supporting a One Health Perspective on Staphylococcus aureus Bovine Mastitis
Source: Antibiotics (Basel). 2026 Jan 18;15(1):98. doi: 10.3390/antibiotics15010098 (PMC12837917; doi:10.3390/antibiotics15010098)
Supplement: Supplementary file 1 [file antibiotics-15-00098-s001.zip › Supplementary_S4.pdf]

Supplemental figure S4: Pan-genome architecture and gene cluster dynamics of 50 H_ *S. aureus* genomes analyzed with PGAP2.

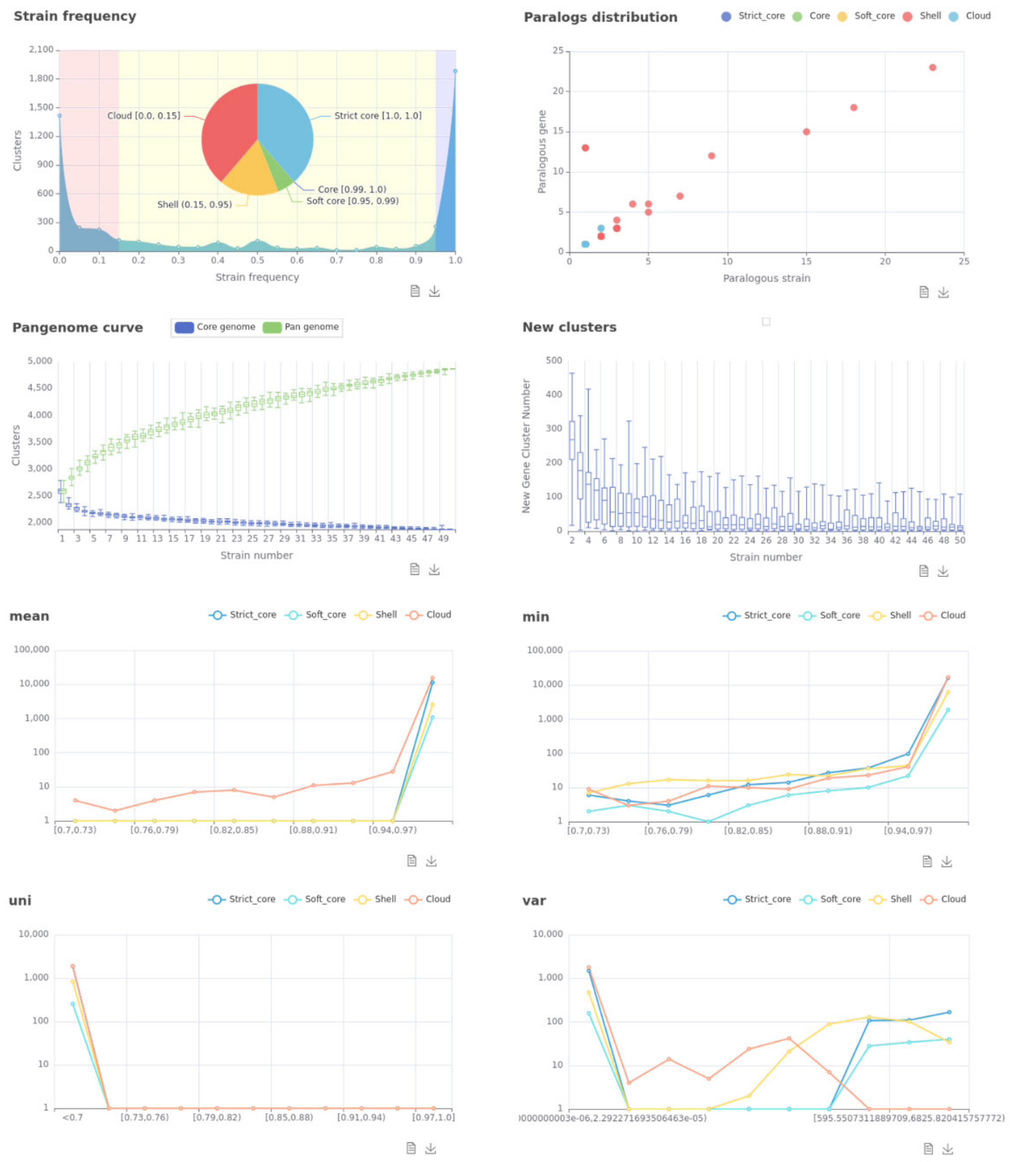

The strain-frequency plot shows that gene clusters are spread out across strict core, core, soft core, shell, and cloud classes. Strict core and core clusters are in the low thousands, while cloud clusters are in the low-frequency range. This means that there is a compact conserved backbone and a large accessory gene pool. The pan- and core-genome curves show that the total number of clusters goes up from about 3,000–3,500 in the first genomes to about 4,500–5,000 when all 50 genomes are included. The core genome, on the other hand, goes down to about 2,000–2,500 clusters, which is in line with an open pan-genome and a stable core. The paralog distribution shows that up to 20–25 strains might have paralogs in a certain frequency class. This shows that gene duplication impacts both conserved and auxiliary parts. Lastly, the "new cluster" and summary statistics (min, unique, variance) broken down by frequency class show that early genomes add several hundred new clusters, while later genomes add fewer and fewer, mostly in the shell and cloud. This shows that the gene repertoire in this population is still growing, but at a slower rate.
